# ﻿Common but ignored: a new species of *Cyrtodactylus* (Chordata, Reptilia, Squamata, Gekkonidae) from lowland Sumatra Barat, Indonesia

**DOI:** 10.3897/zookeys.1169.98681

**Published:** 2023-07-06

**Authors:** Fitra Arya Dwi Nugraha, Yuni Ahda, Djong Hon Tjong, Nia Kurniawan, Awal Riyanto, Muhammad Alif Fauzi, Si-Min Lin

**Affiliations:** 1 Department of Biology, Faculty of Mathematics and Natural Sciences, Universitas Negeri Padang, Jl. Hamka, Kota Padang 25132, Sumatra Barat, Indonesia Universitas Negeri Padang Padang Indonesia; 2 Department of Biology, Faculty of Mathematics and Natural Sciences, Universitas Andalas, Limau Manis, Pauh, Kota Padang 25175, Sumatra Barat, Indonesia Universitas Andalas Padang Indonesia; 3 Department of Biology, Faculty of Mathematics and Natural Sciences, Universitas Brawijaya, Jl. Veteran 65145, Ketawanggede, Lowokwaru, Kota Malang, Jawa Timur, Indonesia Universitas Brawijaya Kota Malang Indonesia; 4 Herpetology Group Research, Biosystematic and Evolution Research Center – National Research and Innovation Agency (BRIN)-(“Museum Zoologicum Bogoriense”), Jakarta, Indonesia Herpetology Group Research, Biosystematic and Evolution Research Center - National Research and Innovation Agency (BRIN)-("Museum Zoologicum Bogoriense") Jakarta Indonesia; 5 Department of Life Science, National Taiwan Normal University, Taipei, Taiwan National Taiwan Normal Universit Taipei Taiwan

**Keywords:** Bent-toed gecko, diversity, morphology, ND2 gene, phylogeny, Sumatra, taxonomy

## Abstract

The lowland region of Sumatra Barat has received little attention in previous biodiversity studies. Past studies have mainly focused on highland habitat and conservation areas. However, many populations of *Cyrtodactylus* in the lowland habitats of Sumatra Barat were not correctly identified. A phylogenetic tree based on the NADH dehydrogenase subunit 2 (ND2) gene showed that the lowland Sumatran population is the sister group of the Malaysian lowland species, *C.semenanjungensis*, together nesting within the *agamensis* group. The genetic divergence within the Sumatra Barat population is 0–4.2% and 18.3–20% to *C.semenanjungensis*. Further examination of morphological characters revealed that they differed from the sister clade and other Sumatran *Cyrtodactylus* members by a unique combination of characters such as absence of tubercle on brachium, presence of tubercle on ventrolateral fold, 32–41 paravertebral tubercles, 38–46 ventral scales, enlarged femoral scales, presence of precloacofemoral pores and 22–23 subdigital lamellae under fourth toe. Based on the morphological and molecular evidence, the lowland Sumatran population is herein described as a new species, increasing the number of species in Sumatra to seven. More comprehensive and intensive sampling efforts would most likely yield further discoveries in the group of Sumatran *Cyrtodactylus* in the near future.

## ﻿Introduction

Species diversity and new discoveries in the Bent-toed gecko genus *Cyrtodactylus* Gray, 1827 is remarkable in recent years. In only two decades from 2000, the number of new species descriptions has dramatically increased this taxon from only 77 species to nearly 300. This steep upward trajectory has indeed indicated that the true diversity of *Cyrtodactylus* is highly underestimated. Among Southeast Asian countries, Indonesia is ranked fifth as the largest contributor (considering type localities) to the number of Bent-toed gecko discoveries, behind Myanmar, Vietnam, Malaysia and Thailand (Grismer et al. 2021). Despite having a larger area than Peninsula Malaysia, the number of species discovered in Sumatra island was significantly lower (6 vs 26), presumably suggesting a low sampling or research effort undertaken on the island. But, perhaps the most influencing factor contributing to its diversity is the evolution of habitat preferences where some habitat transitions have occurred. The changes in habitat preference may in part be responsible for its diversity (Grismer et al. 2020).

Currently, there are six species of *Cyrtodactylus* distributed across the mainland of Sumatra (Teynie et al. 2010; Uetz et al. 2022). Four species were described based on Sumatran type series: *C.agamensis* (Bleeker, 1860); *C.psarops* Harvey, O’Connell, Barraza, Riyanto, Kurniawan & Smith, 2015; *C.semicinctus* Harvey, O’Connell, Barraza, Riyanto, Kurniawan & Smith, 2015; and *C.lateralis* (Werner, 1896). Meanwhile, *C.consobrinus* (Peters, 1871) dan *C.quadrivirgatus* Taylor, 1962 were described based on non-Sumatran type series.

Sampling efforts for herpetofauna specifically targeting the Sumatra Barat region have not been comprehensively performed until the last decade. Teynie et al. (2010) carried out sampling in Sumatra Barat Province and Jambi Province but the majority was in the surroundings of Lake Maninjau. Although they found a significant number of reptiles and amphibian and reported a new species of Bufonidae (Amphibia: Anura), no *Cyrtodactylus* were collected. Milto and Bezman-Moseyko (2021) also surveyed the surroundings of Lake Maninjau, reporting two new records *Limnonectesdeinodon* Dehling, 2014, Dicroglossidae and *Sphenomorphusscotophilus* (Boulenger, 1900) and one new species *Cnemaspiscalderana* Milto & Bezman-Moseyko, 2021 for Sumatra and provided extended descriptions of *Cyrtodactylusagamensis*. Nugraha et al. (2022) surveyed lowland habitat generally close to human settlement such as paddy fields, peat swamp near the coastline, streams and other plantations. They reported *Cyrtodactylus* sp. from a rocky stream and did not provide a description. Unfortunately, there was no sampling effort that focused on collecting lowland *Cyrtodactylus* from Sumatra.

Several potentially new species from Sumatra have been reported by O’Connell et al. (2019). At least four potentially new species among the *C.psarops* group await formal description. We excluded *Cyrtodactylusmarmoratus* Gray, 1831 from Sumatra as this species only occurs in Java (O’Connell et al. 2019) and morphometric assessment of specimens labelled as *C.marmoratus* showed that they did not belong to this species (Fauzi et al. 2022).

Between 2020 and 2022, we surveyed lowland forest in four locations of Sumatra Barat targetting *Cyrtodactylus*. We found several individuals of *Cyrtodactylus* and examined those specimens using both morphological and molecular approaches. Based on our examination on those individuals, we found they differed from other *Cyrtodactylus* in Sumatra and they should be treated as a new species.

## ﻿Materials and methods

### ﻿Field survey and specimen preservation

**Figure 1.**
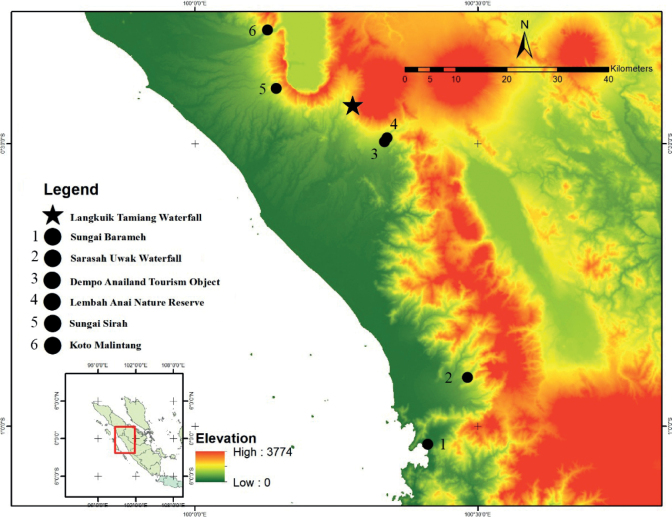
Localities of samples used in this study. The star indicates the type locality. The black-filled circle indicates the locations of the paratypes.

A total of 16 individuals from lowland of Sumatra Barat Province were collected by hand during fieldwork in 2020–2022 (Fig. 1). All Individuals were collected during the night from 19.00 to around 24.00. The locality of samples were recorded using a Garmin GPS 65 s. The collected specimens were euthanized by immersion in 250 mg/L benzocaine solution. The fresh tissues (liver) were preserved in 96% ethanol and stored at -20 °C for genetic analysis, and the specimens were fixed using 10% formalin and then transferred to 70% alcohol for long term preservation. Photographs were taken before and immediately after euthanization using a mirrorless camera Sony Alpha 6000 with Laowa 65 mm f/2.8 macro lens. In order to document the original coloration, we defered documentation until the next day if the individuals showed stress coloration. All photos were deposited at the Department of Biology, Universitas Negeri Padang (UNP) along with the preserved specimens.

### ﻿Morphological analysis

Color characters were assessed from digital images of living individuals prior to preservation. The sex was determined by confirming the presence of the hemiphenal structure by injecting formaline into the postcloacal region. The measurements were taken with a dial caliper to the nearest 0.05 mm under a Nikon SMZ1270 stereo microscope. We followed Grismer and Leong (2005) for examination of morphological and meristic characters including:

**SVL** Snout-vent length, measured from tip of snout to vent;

**AX** Axial length, measured from posterior margin of forelimb insertion to anterior margin of hind limb insertion;

**HL** Head length, measured from tip of snout to articulation of quadrate bone;

**HW** Head width, measured at widest part of head;

**HH** Head height, measured from occiput to underside of lover jaw;

**SL** Snout length, measured from tip of snout to anterior margin of orbit;

**OEL** Orbit-ear length, measured from posterior margin of eye to anterior margin of ear opening;

**OD** Orbit diameter, measured from anterior to posterior margin of orbit;

**EL** Ear length, measured from anterior to posterior margin of ear opening;

**ML** Mental length, maximum length of mental shield;

**EN** Eye-nostril distance, measured from anterior margin eye to posterior margin of external nares;

**IN** Internarial distance, measured between the nares across the rostrum;

**FL** Forearm length, measured from base of palm to elbow;

**TBL** Tibia length, measured from base of heel to knee;

**TaL** Tail length, measured from the vent to the tip of the tail, original or regenerated;

**TaW** Tail width, measured at widest part of tail.

Merisitic charatcers were counted on both right and left sides when possible. The characters were recorded as follows:

**DTR** Dorsal tubercles, number of tubercle rows on dorsum at midbody, counted in one row between lateral folds;

**PVT** Paravertebral tubercles, number of tubercles counted in a longitudinal row between posterior insertion of fore limb and anterior insertion of hind limb;

**VS** Ventral scales, number of ventral scales at midbody, counted in one row between lateral folds;

**EPFS** Enlarged precloaca and femoral scales, number of enlarged precloacofemoral scales, counted along lowest, pore-bearing row;

**PFP** Precloaca and femoral pores, number of precloaca and femoral pores;

**PCT** Postcloacal tubercles, number of postcloaca tubercles, right and left;

**LT4** Subdigital lamellae under 4^th^ toe, subdigital scales under 4^th^ toe, counted from first enlarged scale (true lamellae) on lower side of toe to scale proximal to apical scale;

**SL** Supralabial scales, labial scales of upper jaw, beginning with first enlarged scale bordering rostral shield, ending with last enlarged scale bordering labial angle for right and left side;

**IL** Infralabial scales, labial scales of lower jaw, beginning with first scale bordering mental shield, ending with last enlarged scale bordering labial angle for right and left side;

**IN** Internasal scales, number of scales between rostronasals, bordering rostral shield;

**GUL** Gular scales, number of gular scales bordering pair of 1^st^ postmentals (excluding enlarged second 2^nd^ postmentals)

To make clear the counting of some scales (supra and infralabials, precloacafemoral scales, gular scales) and detection of the presence of pores, we used a staining technique with methylene blue in 70% alcohol (Harvey et al. 2015). For species comparison, we used the published descriptions of other species.

### ﻿Laboratory protocols

Total genomic DNA was extracted from liver tissues using the Qiagen Dneasy tissue kit (Valencia, CA, USA) following the standard protocol for animal tissues. The Natrium Dehydrogense Subunit 2 (ND2) gene and partial flanking tRNAs was amplified by using polymerase chain reactions (PCRs) under the following conditions: a cycle of 9 min at 94 °C, then followed by 35 cycles of 45 s at 94 °C, 45 s at 60 °C and 1 min at 72 °C with a final extension step of 6 min at 72 °C. Amplifications were carried out in 25-µl volume consisting of 2.5 µl genomic DNA (approximately 100 ng), 0.4 µм each primer and 1× GoTaq Green Master Mix (Promega, Wisconsin, USA). The primers used in this study followed Oliver et al. (2012): M112F (5’- AAGCTTTCGGGGCCCATACC-3’) and M1123R (5’- GCTTAATTAAAGTGTYTGAGTTGC -3’). The PCR products were visualized using 1.5% agarose gel for confirmation of PCR amplification. The PCR product was then sent to the sequencing service 1^st^ BASE Singapore (https://base-asia.com/) through PT. Genetika Science Indonesia. The PCR primers were also used for sequencing.

### ﻿Phylogenetic reconstruction

Sequences were uploaded, assembled and editted in Geneious software 2022.2.2 (http://www.geneious.com/). The protein coding-region of the sequences were aligned and translated to amino acid to verify that the targeted sequences were assembled correctly. After that confirmation, all sequences were submitted to GenBank. We followed Grismer et al. (2021) for the classification of species groups. For comparison, ND2 sequences from the *C.darmandvillei*, *C.marmoratus*, *C.lateralis*, *C.sworderi* and *C.agamensis* groups that were available in GenBank were used for phylogenetic tree reconstruction with our samples (Table 1). Phylogenetic relationship was constructed using maximum likelihood (ML) analysis performed using RaxML HPC Black Box with 1000 bootstrap replicates implemented in CIPRES Sience Gateway portal (Miller et al. 2010; accessed through https://www.phylo.org/). We used the GTR+Γ model of sequence evolution. Nodal support with bootstrap (BS) values ≥ 70 was considered as strongly supported (Hillis and Bull 1993). The tree resulted from RAxML was then visualized and editted in iTOL v6 (Letunic and Bork 2021; available at https://itol.embl.de/) and further improvement in Photoshop C6 64-bit. We also calculated uncorrected p-distances using MEGA 7 (Kumar et al. 2016).

**Table 1.** Species used in the phylogenetic reconstruction including localities and GenBank accession numbers of the mitochondrial NADH dehydrogenase subunit 2 gene. PM = Peninsular Malaysia; Gn.= Gunung.

Species	Locality	Museum number	Accession number	Source
***agamensis* group**				
*Cyrtodactylusgonjong* sp. nov.	Agam, Sumatra Barat, Indonesia	UNP193	OR208777	This study
*Cyrtodactylusgonjong* sp. nov.	Agam, Sumatra Barat, Indonesia	UNP194	OR208778	This study
*Cyrtodactylusgonjong* sp. nov.	Agam, Sumatra Barat, Indonesia	UNP199	OR208779	This study
*Cyrtodactylusgonjong* sp. nov.	Agam, Sumatra Barat, Indonesia	UNP203	OR208780	This study
*Cyrtodactylusgonjong* sp. nov.	Agam, Sumatra Barat, Indonesia	UNP146	OR208781	This study
*Cyrtodactylusgonjong* sp. nov.	Tanah Datar, Sumatra Barat, Indonesia	UNP061	OR208782	This study
*Cyrtodactylusgonjong* sp. nov.	Padang, Sumatra Barat, Indonesia	UNP053	OR208783	This study
*Cyrtodactylusgonjong* sp. nov.	Padang, Sumatra Barat, Indonesia	UNP055	OR208784	This study
*Cyrtodactylusgonjong* sp. nov.	Tanah Datar, Sumatra Barat, Indonesia	UNP062	OR208785	This study
*Cyrtodactylusgonjong* sp. nov.	Padang Pariaman, Sumatra Barat, Indonesia	UNP045	OR208786	This study
*Cyrtodactylusgonjong* sp. nov.	Padang Pariaman, Sumatra Barat, Indonesia	UNP047	OR208787	This study
*Cyrtodactylusgonjong* sp. nov.	Padang Pariaman, Sumatra Barat, Indonesia	UNP048	OR208788	This study
*Cyrtodactylusgonjong* sp. nov.	Padang, Sumatra Barat, Indonesia	UNP053	OR208789	This study
*Cyrtodactylusgonjong* sp. nov.	Tanah Datar, Sumatra Barat, Indonesia	UNP060	OR208790	This study
*Cyrtodactylusgonjong* sp. nov.	Padang Pariaman, Sumatra Barat, Indonesia	UNP165	OR208791	This study
*Cyrtodactylusgonjong* sp. nov.	Padang Pariaman, Sumatra Barat, Indonesia	UNP167	OR208792	This study
*C.metropolis*	Batu caves, Selangor, PM	LSUHC 11343	KU253579	Grismer et al. 2016
*C.payacola*	Bukit Panchor, Penang, PM	LSUHC 10070	JQ889190	Johnson et al. 2012
*C.majulah*	Nee Soon Swamp, Singapore	ZRC 26951	JX988529	Grismer et al. 2013
*C.pantiensis*	Gn. Panti, Johor, PM	LSUHC 8905	JQ889186	Johnson et al. 2012
*C.tiomanensis*	Pahang, PM	LSUHC 6251	JX440563	Wood et al. 2012
*C.rosichonariefi*	Bunguran, Great Natuna, Indonesia	MZB Lace 12132	KP256187	Riyanto et al. 2015
*C.psarops*	Indonesia	MZB 9687	MH248931	O’Connell et al. 2019
*C.* sp. 3	Indonesia	ENS 18140	MH248911	O’Connell et al. 2019
*C.* sp. 4	Indonesia	ENS 18591	MH248912	O’Connell et al. 2019
*C.* sp. 5	Indonesia	ENS 18659	MH248916	O’Connell et al. 2019
*C.* sp. 6	Indonesia	ENS 18719	MH248917	O’Connell et al. 2019
*C.semenanjungensis*	Gn. Panti, Johor, PM	LSUHC 8900	JQ889177	Johnson et al. 2012
*C.semicinctus*	Indonesia	ENS 14749	MH248925	O’Connell et al. 2019
C.cf.agamensis	Indonesia	ENS 19636	MH248910	O’Connell et al. 2019
C.cf.agamensis	Indonesia	ENS 19635	MH248909	O’Connell et al. 2019
C.cf.agamensis	Indonesia	ENS 19634	MH248908	O’Connell et al. 2019
C.cf.agamensis	Indonesia	ENS 19694	MH248907	O’Connell et al. 2019
***sworderi* group**				
*C.quadrivirgatus*	Bukit Larut, Perak, PM	LSUHC 8859	JQ889241	Johnson et al. 2012
*C.guakanthanensis*	Gua Kanthan, Perak, PM	LSUHC 11323	KU253577	Grismer et al. 2016
*C.tebuensis*	Gn. Tebu, Terengganu, PM	LSUHC 10902	JX988527	Wood et al. 2012
*C.sworderi*	Sungai Kawal, Peta, PM	LSUHC 7685	JQ889189	Johnson et al. 2012
*C.gunungsenyumensis*	Hutan Lipur Gn. Senyum, Pahang, PM	LSUHC 12201	KU253585	Grismer et al. 2016
***lateralis* group**				
*C.lateralis*	Indonesia	UTA 62916	KU893163	Harvey et al. 2016
*C.rubidus*	–	CES 131445	KM255203	Agarwal et al. 2014
*C.durio*	Malaysia	LSUHC 9725	KU893159	Harvey et al. 2016
***marmoratus* group**				
*C.marmoratus*	Indonesia	ENS 15932	KR921721	Harvey et al. 2015
*C.papuensis*	–	SAMA R62652	JQ820320	Oliver et al. 2012
*C.* sp. 1	Indonesia	ENS 15813	KR921697	Harvey et al. 2015
*C.* sp. 2	Indonesia	ENS 15784	KR921689	Harvey et al. 2015
***darmandvillei* group**				
*C.batucolus*	Pulau Besar, Melaka, PM	LSUHC 8934	JQ889179	Johnson et al. 2012
*C.petani*	Pasuruan, Jawa Timur, Indonesia	MZB Lace 11706	KU232620	Grismer et al. 2016
*C.kimberleyensis*	Siuna, Sulawesi Tengah, Pulau Sulawesi, Indonesia	WAM R164144	JX440544	Wood et al. 2012
*C.jellesmae*	Siuna, Sulawesi Tengah, Pulau Sulawesi, Indonesia	RMB 1672	GU550721	Siler et al. 2010
*C.sadleiri*	Christmas island, Australia	SAMA R34810	JQ820309	Oliver et al. 2012
*C.seribuatensis*	Pulau Nangka Kecil, Johor, PM	LSUHC 6349	JQ889187	Johnson et al. 2012
*C.darmandvillei*	Nusa Tenggara Barat, Indonesia	WAM R98393	JX440533	Wood et al. 2012
**Outgroup**				
*Hemidactylusfrenatus*	–	LLG 4871	GQ458049	Murthy et al. 2015
*Gekkogecko*	Thailand: Patong Beach, Kathu District, Phuket Island, Phuket Province	MVZ 215314	AF114249	Macey et al. 1999

## ﻿Results

### ﻿Phylogenetic relationships of *Cyrtodactylus* from Sumatra Barat

**Figure 2.**
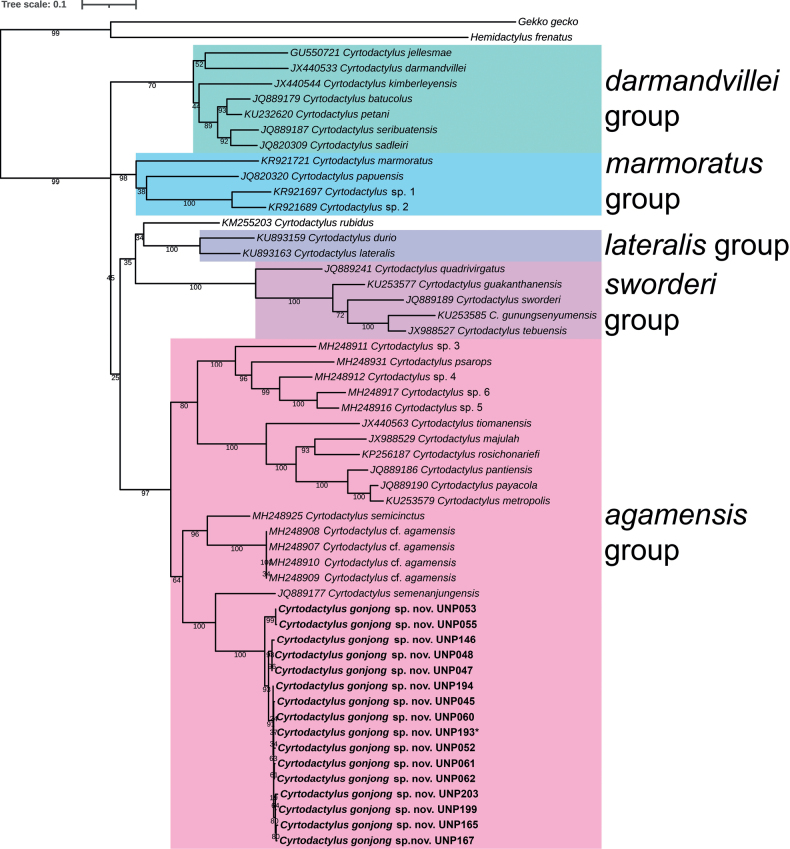
The maximum likelihood (ML) tree topology of the new species with other *Cyrtodactylus* inferred by the ND2 gene sequences. The light orange box indicates the *C.agamensis* group members (the sample indicated by an asterisk denotes the holotype). The numbers on the branches are bootstrap values.

After assembling sequences, we obtained 609 to 1027 basepairs of ND2 gene for subsequent alignment and phylogenetic tree construction. In general, the ML tree topology was congruent with a previous study (Grismer et al. 2021) in term of inter-relationship among major groups; i.e., *C.agamensis*, *C.sworderi*, *C.lateralis*, *C.marmoratus* and *C.darmandvillei*. We obtained a new putative species that was strongly supported as a sister clade to *Cyrtodactylussemenanjungensis* Grismer & Leong, 2005 from Peninsular Malaysia (BS = 100). Together they nested within the *C.agamensis* group (Grismer et al. 2021) along with *C.semicinctus*, *C.psarops* and other non-Sumatran species (e.g., *Cyrtodactyluspayacola* Johnson, Quah Anuar, Muin, Wood, Grismer, Greer, Onn, Ahmad, Bauer & Grismer, 2012 and *Cyrtodactylusmajulah* Grismer, Wood & Lim, 2012) (Fig. 2). The genetic distance within the new putative species group was 0–4.2% (mean = 1.7%) and the distance between them to *C.semenanjungensis* was 18.3–20% (mean = 19.3%). The p-distance of new putative species to other species is more than 17% (Table 2). We herein describe this population as new to science. Although the bootstrap value separating the new putative species from *C.semenanjungensis* is significant, the bootstrap value between the new putative species and other *agamensis* group species (*C.semenanjungensis*, *C.semicinctus*, C.cf.agamensis) is less than 70.

**Table 2.** Uncorrected pairwise genetic distance (%) within the new putative species and with the closely related lineages based on the ND2 gene.

No.	Species	1	2	3	4
1.	*C.* sp.	0–4.2			
2.	*C.semenanjungensis*	18.3–20.0			
3.	*C.semicinctus*	18.7–19.7	18.6		
4.	C.cf.agamensis	24.6–26.0	27.6	17.6	
5.	*C.psarops*	31.9–38.4	31.9	27.3	30.5

### ﻿Taxonomy

#### ﻿ Cyrtodactylus gonjong sp. nov.

Taxon classificationAnimaliaSquamataGekkonidae

DD6147C0-25CE-5190-98ED-4EE7EA7A0E53

https://zoobank.org/EFDA2781-5260-43A7-9A59-B5BBE26D5348

##### Type material.

**Figure 3.**
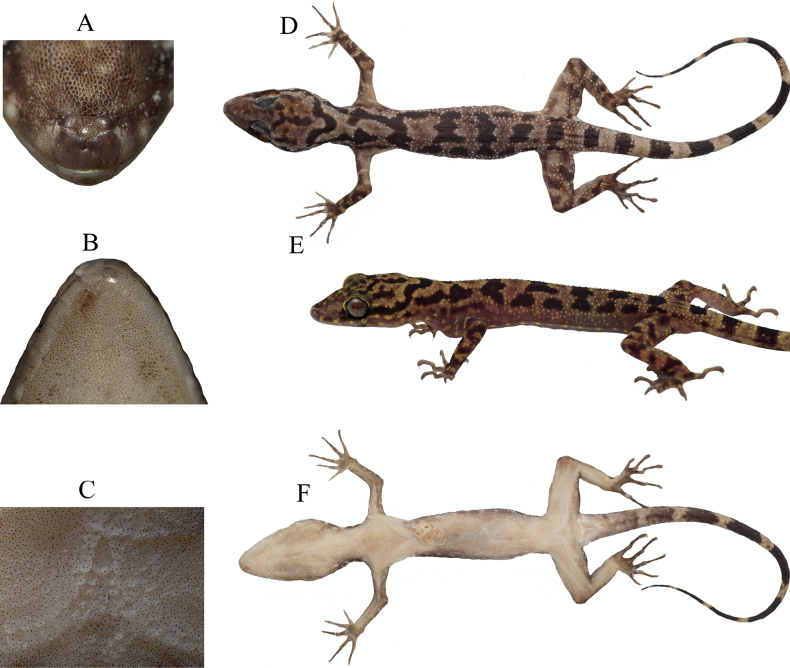
The holotype (UNP193, adult female, SVL 65.1 mm) **A** rostral view **B** gular view **C** precloacal scales **D** dorsal **E** ventrolateral **F** ventral view. **A–D, F** were taken after 1 month preservation **E** is a live individual.

**Figure 4.**
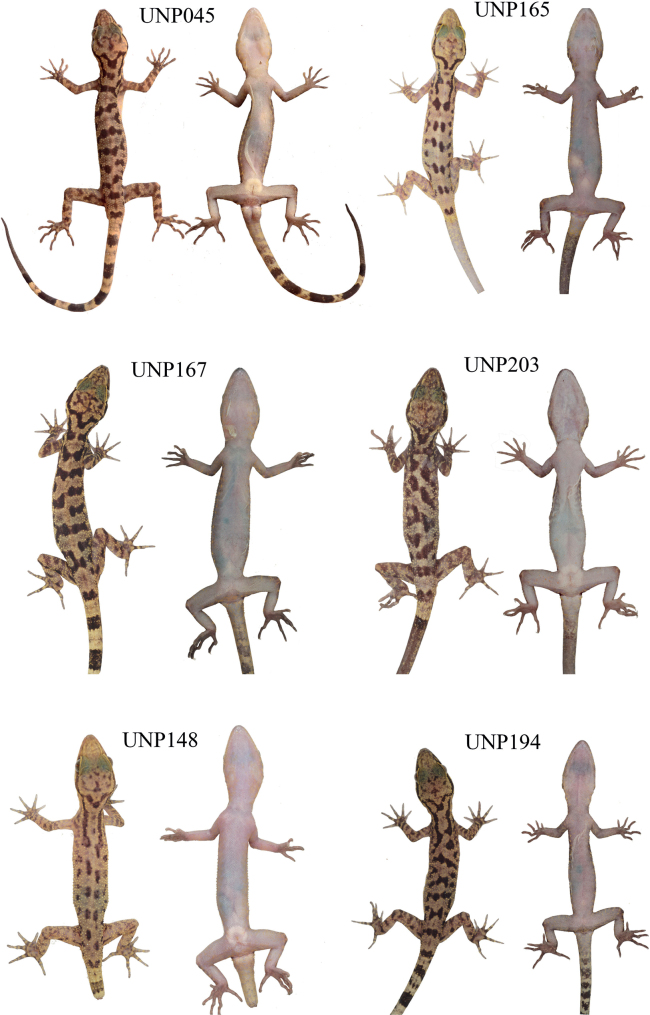
The representative paratypes of *C.gonjong* sp. nov.

***Holotype*.** UNP193 (Fig. 3) adult female, collected from Langkuik Tamiang waterfall (0°26'0.7404"S, 100°16'44.5404"E), Nagari (Village) Malalak Selatan, Kecamatan (District) Malalak, Kabupaten (Regency) Agam, Sumatra Barat Province, Indonesia, on 22 July 2022 by Fitra Arya Dwi Nugraha (FADN), Yunico Amardi (YA) and Mahesa Rafi. ***Paratypes*** (Fig. 4). One adult male (UNP045) collected from Anai Dempoland tourism object (0°29'46.8312"S, 100°20'7.638"E), village Kayu Tanam, district 2×11 Kayu Tanam, regency Padang Pariaman, Sumatra Barat Province on 3 June 2020 by FADN and YA. Two adult males (UNP052, UNP053) collected from village Sungai Barameh (1°1'54.71"S, 100°24'43.69"E), district Padang Selatan, Padang city, Sumatra Barat Province on 31 January 2021 by FADN and YA. One adult male (UNP060) collected from Lembah Anai Nature Reserve (0°29'24"S, 100°20'24"E), district X Koto, regency Tanah Datar, Sumatra Barat Province on 10 June 2021 by Mahesa Rafi, Katon Agusdi, Fadhil Raid and Fachrul Rozi Octavian. One adult male (UNP148) collected from Sarasah Uwak Waterfall (0°54'28"S, 100°28'54"E), village Limau Manis, district Pauh, Padang city, Sumatra Barat Province on 31 May 2022 by FADN. Two adult females (UNP165, UNP167) collected from village Sungai Sirah (0°24'8.8128"S, 100°8'37.6728"E), district Sungai Geringging, regency Padang Pariaman, Sumatra Barat Province on 7 June 2022 by FADN and YA. One juvenile (UNP194), same data as holotype. Three adult males (UNP196, UNP199, UNP203) collected from village Koto Malintang (0°17'56.3748"S, 100°7'42.5064"E), district Tanjung Raya, regency Agam, Sumatra Barat Province.

##### Etymology.

The specific ephitet *gonjong* is taken from the name of the roof style of the typical house in Sumatra Barat created by its ethnic people, called *Minang*. The house itself, called *Rumah Gadang*, and the unique style of the roof shape was inspired by the horn of buffalo, the most respected animal in Minang ethnology. The *gonjong* has become a symbol used by the Minang people to show their ethnic identity outside Sumatra Barat and now is used not only for the house but also for government buildings, restaurant buildings, hotels or other public venues. This name is given as an honour to the Minang people because they were helpful during our survey.

##### Diagnosis.

*Cyrtodactylusgonjong* sp. nov. is assigned to the *C.agamensis* group on the basis of its recovered phylogenetic position (Fig. 2). This species can be differentiated from all congeners within the *C.agamensis* group by having the following combination of characters: (1) a medium-sized *Cyrtodactylus*, 54.1–77.7 mm in adult males, 65.1–76.7 mm in adult females; (2) 10–13 enlarged supralabial and 9–13 enlarged infralabial scales; (3) 3–5 internasal scales; (4) antebrachium tuberculated, brachium not tuberculated; (5) 32–41 paravertebral tubercles; (6) 16–19 longitudinal rows of dorsum tubercles; (7) 38–46 ventral scales; (8) 22–24 subdigital lamellae on the fourth toe; (9) 34–46 enlarged femoroprecloacal scales; (10) femoroprecloacal pores present in both sexes, 13–36 in adult males, 0–18 in adult females; (11) precloacal groove or depression present; (12) enlarged median subcaudals absent; (13) distinct ventrolateral folds; (14) subconical prominent tubercles on body that extend to the base of the tail; (15) 2–3 postcloacal tubercles; (16) two postocular stripes fused to a U-shaped mark on occiput; (17) postocular stripes extended beyond the arms; (18) 7–8 dark bands on trunk; (19) tail ringed by beige and black; and (20) labials yellow or dark with yellow/beige spots.

##### Description of holotype.

Adult female with 65.1 mm SVL; head moderately in length (HL/SVL 0.29), wide (HW/HL 0.60), slightly flattened (HH/HL 0.34), distinct from neck, and triangular shape from dorsal view; lores concave; canthus rostralis rounded; snout elongated (SL/HL 0.40), rounded in rostral region, eye to snout distance larger than head depth; eyes large (OD/ HL 0.22), obtrusive and appeared beyond labials in dorsal view, eye diameter less than the eye to ear distance, pupil vertical; ear oppening small (EL/HL 0.03), elliptical, oriented obliquely leaning posteriorly; rostral large, subrectangular in shape, medial posterior edge interupted by an subhexagonal internasal scale that embedded within rostral, posteriorly bordered by three internasal scales, laterodorsally by nostril opening and lateroventrally by first supralabial; external nares directed lateroposteriorly, bordered anteriorly by rostral, posteriorly by two postnasals: one subcircular, one crescent shaped, dorsally by large supranasal, and ventrally by first supralabial; supranasal subrectangular, separated by one subhexagonal scale and a smaller subrectangular scale that piled up, the smaller one above the subhexagonal one, supranasal laterally bordered by nostril, right supranasal anteriorly bordered by five significantly smaller scales, left supranasal anteriorly bordered by four significantly smaller scales; one internasal scale, subhexgonal in shape, embedded within rostral, bordered by two supranasal scales, one smaller scale right posteriorly, and rostral anteriorly; 9/10 (right/left) supralabial scales to below center of the eye, 10/11 (right/left) to the posteriormost enlarged scale, 8 infralabial scales to below center of the eye, 10/11 (right/left) to the posteriormost enlarged scale; scales of frontonasal, prefrontal and lores small, juxtaposed, relatively raise; weak tubercles on the supraorbital region; prominent tubercles above the ear opening, larger than those on supraorbital, the tubercles gradually increased in size posteriorly.

Body slender, relatively short (AX/SVL 0.47), with distinct ventrolateral fold; scales on dorsum small, homogenous, interspersed by rounded to trihedral tubercles; tubercles present from the occiput region to the base of the tail but no further than 1/3 of the tail, being more dense gradually posteriorly until the base of the tail; 37 paravertebral tubercles; 17 tubercles transversally in the middle of the trunk; imbricate and smooth ventral scales, scales in middle larger than those on lateral and dorsal, 42 scales across the center of the trunk from one ventrolateral fold to another; femoral scales enlarged, extending until 2/3 portion of femora, contiguous with enlarged precloacal scales, forming a reverse V-shaped mark in the middle of hindlimb; a greatly enlarged scale at the apex, larger than other precloacal and femoral scale, rather long than wide, each right and left bordered by three smaller scales; 46 continuous enlarged precloacal and femoral scales, all similar in size except the scale at apex; precloacal groove or depression absent.

Limbs moderately slender; forelimbs relatively short (FL/SVL 0.15); scales on forelimbs dorsum larger than on body dorsum, rostrum or frontal, domed to subconical in shape, scales near the junction of limbs with trunk being smaller; round to subconical tubercles present on antebrachium, concentrated in the middle of antebrachium, similar in size with those on nape; brachium not tuberculated; fingers relatively long, well developed, no webbing; claw well developed, relatively short; hind limbs more robust than fore limbs, moderate in length (TBL/SVL 0.19); scales on dorsum domed to subconical, interspersed with trihedral tubercles; tubercles on dorsum approximately similar in size with those on posterior trunk; anterior ventral scales small, gradually increase in size posteriorly, the scales after enlarged femoral scales much smaller than those on anterior part; 22 subdigital lamellae on fourth finger; toes relatively long, claw well developed, relatively short.

Tail 78.9 mm in length, longer than SVL (TL/SVL 1.21), 5.4 mm in width at base, cylindrical, decreasing in size posteriorly; scales on tail dorsum small, approximately similar in size with those on the distal of femoral; tail dorsum tuberculated at its base, no more than 1/3 of tail; postcloacal tubercles at each side, left tubercles slightly searated, right tubercles in contact one another; no enlarged median subcaudal scale; subcaudal scales larger than on tail dorsum.

##### Coloration in life.

Dorsal ground color of head, neck, trunk, limbs and tail beige to weak yellow; yellow mottling along the ventrolateral fold, on the lateral part of neck, on supra and infralabial scales; ventral of head, neck, trunk, limbs whitish to grey pale; palmar, metatarsal, fingers and toes darker; basal subcaudal ground color whitish to pale grey with dense yellow mottling; most of the original tail except basal subcaudal encircled by beige and black; labials yellow or dark with beige or yellow spots; weak black stripe between nostril and eye (sometimes absent); lateral stronger black stripe extends from posterior margin of the eye to approximately second black band on dorsum; wide U-shaped band around occiput connects with lateral stripe; the U-shaped band usually continuous (only one exception in UNP165 which is discontinuous in the middle); the Y-shaped pattern on occiput present in two individuals (UNP045, UNP193), the others lack Y-shaped pattern instead of irregular network or spots; bands on trunk dorsum sometimes clearly lying transversally and sometimes blotched; strong nape black spot only present in one individual (UNP045) where left portion fused with lateral stripe, fainted nape black spot presents in two individuals (UNP148, UNP165), the others lack nape spot; black lateral spots present positioned parallel with dorsum bands or between them; 5–6 irregular black bands on forelimbs and hindlimbs; beige to yellow spots on the base fingers (except finger 1); dorsum first finger lighter than other fingers; the clear beige and black bands on subcaudal appear after the one third portion from basal.

##### Comparison.

*Cyrtodactylusgonjong* sp. nov. can be differentiated from all other congeners in the *C.agamensis* group based on a combination of morphological characters.

*Cyrtodactylusgonjong* sp. nov. differs from its most close relative, *C.semenanjungensis* from Peninsular Malaysia by having a larger body size, SVL maximum 77.7 in adult male and 76.7 in adult female (vs 62.1 mm in adult male and 69 mm in adult female); 8–10 supralabial scales to center of eye (vs 11–15); 16–19 DTR (vs 18–20); maximum PVT reachs 41 (vs 37); 38–46 ventral scales (vs 49–53); enlarged femoral scales present (vs absent); femoral and precloacal pores present in both sexes (vs absent in both sexes); 22–24 subdigital lamellae under fourth toe (vs 20–21); a black spot on the nape absent (vs present); posterior end of lateral stripe far beyond the arm insertion to the body, approximately reaching the second band on dorsum (vs between the arms insertion); posterior end of lateral stripe in contact with dorsum band that is separated (vs lateral stripe in contact with clear, not separated band or non-blotched band, creating a box encircled the nape spot); mostly lack nape spot, if present the spot is faint (vs strong black nape spot present).

*Cyrtodactylusgonjong* sp. nov. differs from *C.semicinctus* by being smaller in the adult female (76.7 mm vs 89 mm) and larger in the adult male (77.7 mm vs 75 mm); having fewer DTR (16–19 vs 29–35); having more PVT (32–41 vs 24–27); maximum ventral scales 46 (vs 44); 0–36 precloacal and femoral pores (vs 36–38); 22–24 subdigital lamellae under the fourth toe (vs 19–22);10–13 enlarged supralabial scales (vs 8–11); 10–13 enlarged infralabial scales (vs 8–10); lateral stripe extending from behind the eye to behind arm present (vs absent); labials with beige or yellow spot (vs without spot); beige and black band encircled tail and subcaudal (ringed tail) (vs only dorsum tail – not ringed tail).

*Cyrtodactylusgonjong* sp. nov. differs from *C.psarops* by being smaller in the adult female (76.7 mm vs 82 mm) and larger in the adult male (77.7 mm vs 74 mm); 16–19 dorsal tubercles transversally (vs 28–38); 32–41 longitudinal row tubercles on the middle of the body (vs 23–26); maximum number of ventral scales 46 (vs 49); 0–36 pores on precloacal and femoral region (vs 28–32); 2–3 postcloacal tubercles (vs usually single); 10–13 enlarged supralabials (vs 9–12); 10–13 enlarged infralabial scales (vs 8–11); brachium not tuberculated (vs tuberculated); postocular stripes, left and right, fused to form U-shaped mark on the occiput (vs usually does not fuse to form U-shaped mark); labials usually pale grey or yellow (vs charcoal or dark).

*Cyrtodactylusgonjong* sp. nov. differs from *C.agamensis* by being being smaller in the adult female (76.7 mm vs 86.8 mm) and larger in the adult male (77.7 mm vs 74.9 mm); 10–13 infralabial scales (vs 9–12); maximum number of dorsal tubercles transversally (21 vs 19); maximum number of paravertebral tubercles 41 (vs 37); 38–46 ventral scales (vs 50–67); 13–36 precloacal and femoral pores in males (vs 9–10), 0–18 in females (vs 0–7); 7–8 body black bands on trunk (vs 6–7); postocular stripe extends beyond the arms present (vs absent).

##### Variations.

**Figure 5.**
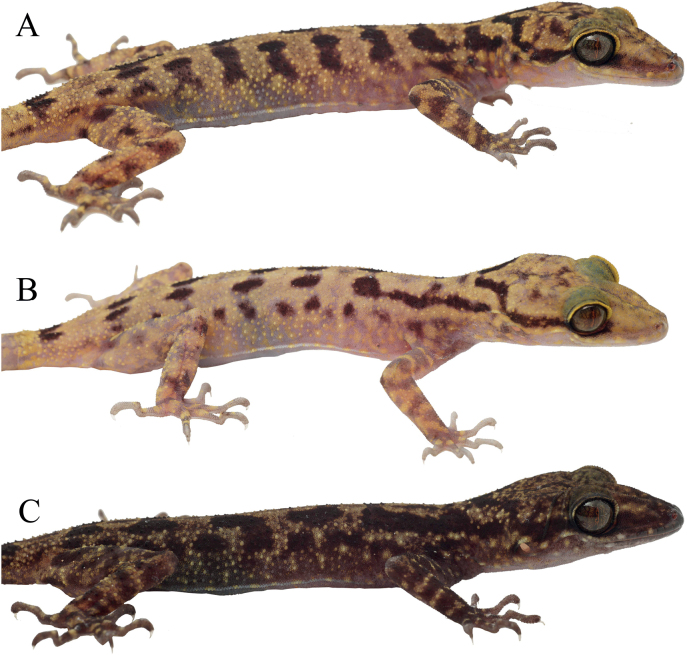
Variation of labial color, stripe between eye and nostril and body bands in lateral view **A** UNP167 **B** UNP165 **C** UNP196.

Dorsal ground color of head, neck, trunk, limbs and tail beige to weak yellow. Labials yellow (UNP148, UNP165, UNP167) or dark on the first three supralabials but pale grey for the rest (UNP193, UNP194, UNP199, UNP203) or dark on first eight supralabials (UNP196) (Fig. 5). Stripe between eye and nostril absent (UNP045, UNP052, UNP060, UNP148, UNP165, UNP193), faint (UNP167, UNP194, UNP199, UNP203), or stronger (UNP196). Lateral stripe that extends beyond the arm could be slightly discontinuous right in the arm insertion (UNP167), while the others are continuous. The medium part of U-shaped mark is discontinuous in UNP165, while the others are continuous (Fig. 4). The bands on the trunk continuous (UNP045, UNP193, UNP167, UNP194, UNP196), while others are irregular (UNP052, UNP199, UNP203) or blotched (UNP060, UNP148, UNP165).

##### Distribution, habitat and natural history.

All individuals were captured in Sumatra Barat Province, from the location between c. 83 meter above sea level (m asl) to c. 700 m asl. In Sungai Barameh, UNP052 was captured when it was sticking on a vertical cement wall approximately 70 cm above the ground; UNP053 was found on a vertical metal roofing sheet which was similar to UNP052 in height; UNP055, a juvenile, was found perching on a fern leave. In Malibo Anai, UNP045, UNP047, UNP048 were found in a similar habitat, sticking on a vertical stem tree approximately 1 m from the ground. In Lembah Anai Nature Reserve, UNP060-062 were found similar to those found in Malibo Anai. However, in Lembah Anai Nature Reserve, we observed an uncollected individual on the forest floor with leaf litter. In Sarasah Uwak Waterfall, we found UNP146 perching on a horizontal branch of a herb approximately 1 m above the ground. In village Sungai Sirah, UNP165 and UNP167 were found perching on a vertical stem of a tree approximately 1 m above the ground. In Langkuik Tamiang waterfall, UNP192 was found on a rock in the wall of a small rocky stream; UNP193 was found on herb branch; and UNP194 was found on a horizontal dead tree approximately 1.2 m above the ground. In village Koto Malintang, we found UNP199 and UNP203 perching on a herb branch approximately 1 m above the ground. There was one female carrying two eggs that can be seen inside the abdomen (UNP165; see Fig. 4).

## ﻿Discussion

We found significant support for separation of the new species from the sister clade. However, our results also showed a low bootstrap (<70) branch between the *C.semicinctus* clade and the *C.semenanjungensis* clade which indicate the topology of this branch remains unstable.

Genetically, the new species is closely related to *C.semenanjungensis* which was supported by the similar morphological characters between them such as the U-shaped mark on occiput, yellow labials, and the brachial tuberculation. Other characters such as the postocular stripe extending beyond the arm and yellow spots on the labials are more similar to *C.psarops*. The greatly enlarged scale at the apex of the pore-bearing series, as mentioned by a previous study (Harvey et al. 2015), is also possessed by *C.gonjong* sp. nov. indicating that this character is a synapomorphy within Sumatran *Cyrtodactylus*. The absence of the enlarged scale at the apex of the pore-bearing series in Malaysian *C.semenanjungensis* is a variation shared between Sumatran species with Malay Peninsular species.

Compared to Malaysia, Myanmar or Vietnam, herpetological research on Sumatra island is far from enough, especially for *Cyrtodactylus* taxa. According to our results, the diversity of Sumatran *Cyrtodactylus* remains unresolved. Despite the additional species of *Cyrtodactylus*, Sumatra still has lower diversity compared to Peninsula Malaysia (7 vs 26). It strongly suggests that the sampling effort has not been sufficient to uncover the diversity. For example, O’Connell et al. (2019) revealed that there are at least six undescribed lineages of *Cyrtodactylus* along Sumatra island. Our study also revealed that even in lowland forest, there is a commonly encountered, but undescribed taxon new to science. This discovery supported the dispersal scenario of *Cyrtodactylus* suggested by O’Connell et al. (2019) in which Sumatra was invaded by lowland species from the Thai-Malay Peninsula (indicated by sister-taxon groupings between *C.gonjong* sp. nov. and *C.semenanjungensis*). Perhaps, the lowland connection occurred during the Miocene which then facilitated dispersal of this species. This discovery also explains O’Connell’s study which predicted lowland species from Sumatra likely exist but were unsampled.

## Supplementary Material

XML Treatment for Cyrtodactylus gonjong

## ﻿Additional information

### Author ORCIDs

Fitra Arya Dwi Nugraha https://orcid.org/0000-0002-0048-8515

Yuni Ahda https://orcid.org/0000-0003-0545-2965

Djong Hon Tjong https://orcid.org/0000-0002-4743-9964

Awal Riyanto https://orcid.org/0000-0002-6887-9352

Muhammad Alif Fauzi https://orcid.org/0000-0003-0975-9681

Si-Min Lin https://orcid.org/0000-0001-7080-706X

### Data availability

All of the data that support the findings of this study are available in the main text or Supplementary Information.
